# A prospective multicentre study in Sweden and Norway of mental distress and psychiatric morbidity in head and neck cancer patients

**DOI:** 10.1038/sj.bjc.6690420

**Published:** 1999-05

**Authors:** E Hammerlid, M Ahlner-Elmqvist, K Bjordal, A Biörklund, J Evensen, M Boysen, M Jannert, S Kaasa, M Sullivan, T Westin

**Affiliations:** Department of Otolaryngology and Head and Neck Surgery, Sahlgrenska University Hospital, Göteborg University, Göteborg, S-413 45, Sweden; Department of Oto-Rhino-Laryngology, Malmö University Hospital, Sweden; Department of Oncology, University Hospital, Trondheim, Norway; Department of Oto-Rhino-Laryngology, University Hospital, Lund, Sweden; The Norwegian Radium Hospital, Department of Medical Oncology and Radiotherapy, Oslo, Norway; Department of Otolaryngology and Head and Neck Surgery, National Hospital, Rikshospitalet, Oslo, Norway; Health Care Research Unit, Institute of Internal Medicine, Sahlgrenska University Hospital, Göteborg University, Sweden

**Keywords:** head and neck cancer, psychiatric morbidity, HAD scale, quality of life

## Abstract

A Swedish/Norwegian head and neck cancer study was designed to assess prospectively the levels of mental distress and psychiatric morbidity in a heterogeneous sample of newly diagnosed head and neck cancer patients. A total of 357 patients were included. The mean age was 63 years, and 72% were males. The patients were asked to answer the HAD scale (the Hospital Anxiety and Depression scale) six times during 1 year. The number of possible or probable cases of anxiety or depression disorder was calculated according to standardized cut-offs. Approximately one-third of the patients scored as a possible or probable case of a major mood disorder at each measurement point during the study year. There were new cases of anxiety or depression at each time point. The anxiety level was highest at diagnosis, while depression was most common during treatment. Females were more anxious than males at diagnosis, and patients under 65 years of age scored higher than those over 65. Patients with lower performance status and more advanced disease reported higher levels of mental distress and more often scored as a probable or possible cases of psychiatric disorder. Our psychometric analyses supported the two-dimensional structure and stability of the HAD scale. The HAD scale seems to be the method of choice for getting valid information about the probability of mood disorder in head and neck cancer populations. The prevalence of psychiatric morbidity found in this study emphasizes the importance of improved diagnosis and treatment. © 1999 Cancer Research Campaign


*British Journal of Cancer* (1999) **80**(5/6), 766–774
© 1999 Cancer Research Campaign
Article no. bjoc.1998.0420

### A prospective multicentre study in Sweden and Norway of mental distress and psychiatric morbidity in head and neck cancer patients

**E Hammerlid[1], M Ahlner-Elmqvist[2], K Bjordal[3,5], A Biörklund[4], J Evensen[5], M Boysen[6], M Jannert[2], S Kaasa[3], M Sullivan[7] and T Westin[1]**

[1]Department of Otolaryngology and Head and Neck Surgery, Sahlgrenska University Hospital, Göteborg University, S-413 45 Göteborg, Sweden; [2]Department of Oto-Rhino-Laryngology, Malmö University Hospital, Sweden; [3]Department of Oncology, University Hospital, Trondheim, Norway; [4]Department of Oto-Rhino-Laryngology, University Hospital, Lund, Sweden; [5]The Norwegian Radium Hospital, Department of Medical Oncology and Radiotherapy, Oslo, Norway; [6]Department of Otolaryngology and Head and Neck Surgery, National Hospital, Rikshospitalet, Oslo, Norway; [7]Health Care Research Unit, Institute of Internal Medicine, Sahlgrenska University Hospital, Göteborg University, Sweden

**Summary** A Swedish/Norwegian head and neck cancer study was designed to assess prospectively the levels of mental distress and psychiatric morbidity in a heterogeneous sample of newly diagnosed head and neck cancer patients. A total of 357 patients were included. The mean age was 63 years, and 72% were males. The patients were asked to answer the HAD scale (the Hospital Anxiety and Depression scale) six times during 1 year. The number of possible or probable cases of anxiety or depression disorder was calculated according to standardized cut-offs. Approximately one-third of the patients scored as a possible or probable case of a major mood disorder at each measurement point during the study year. There were new cases of anxiety or depression at each time point. The anxiety level was highest at diagnosis, while depression was most common during treatment. Females were more anxious than males at diagnosis, and patients under 65 years of age scored higher than those over 65. Patients with lower performance status and more advanced disease reported higher levels of mental distress and more often scored as a probable or possible cases of psychiatric disorder. Our psychometric analyses supported the two-dimensional structure and stability of the HAD scale. The HAD scale seems to be the method of choice for getting valid information about the probability of mood disorder in head and neck cancer populations. The prevalence of psychiatric morbidity found in this study emphasizes the importance of improved diagnosis and treatment.

**Keywords:** head and neck cancer; psychiatric morbidity; HAD scale; quality of life

The psychiatric morbidity among cancer patients has been estimated previously and it has been found that approximately 25–30% of all cancer patients develop clinically significant anxiety or depression within 2 years of diagnosis (Maguire, 1992; Greer, 1994). Several risk factors for development of psychiatric disorder have been identified and examined. These factors relate to patient characteristics, disease and treatment, interaction between patients and illness, and environment (Harrison and Maguire, 1994; Harrison et al, 1994).

Head and neck cancer patients' psychiatric morbidity has been studied in cross-sectional studies (Morton et al, 1984; Espie et al, 1989; Rapoport et al, 1993; Bjordal and Kaasa, 1995; McDonough et al, 1996) and prospectively (Davies et al, 1986; Baile et al, 1992; Hammerlid et al, 1997*a*, 1997*b*). The level of mental distress among these patients corresponds with those found for other cancer locations. The determining risk factors for development of psychiatric morbidity among head and neck cancer patients have been suggested to include tumour stage, performance status, lack of social support and low social functioning, co-morbidity, previous psychiatric disease, pain and malnutrition (Shapiro and Kornfeld, 1987; Westin et al, 1988; Baile et al, 1992; Bjordal and Kaasa, 1995; McDonough et al, 1996; McQuellon and Hurt, 1997), but no single consistently strong risk factor has yet been found.

For this study, a protocol was designed to enable a descriptive, prospective Swedish/Norwegian multicentre quality of life (QL) study of a large sample of head and neck cancer patients accrued during a short period of time to be performed (Bjordal et al, 1993). The study design was tested and proved feasible in a previous study (Hammerlid et al, 1997*a*). The battery of questionnaires found sensitive to change over time in this cancer population included, apart from the Hospital Anxiety and Depression scale (HAD) presented in this paper, the European Organization for Research and Treatment of Cancer Core 30 questionnaire (EORTC QLQ-C30) and the EORTC head and neck cancer module, (QLQ-H&N35). The entire set of questionnaires was presented to 357 newly diagnosed head and neck cancer patients six times during 1 year at different hospitals in Sweden and Norway. Results using the EORTC questionnaire technique will be presented elsewhere (Hammerlid et al, unpublished data; Bjordal et al, unpublished data).

This paper is concerned with the mental distress aspects and presents the results of the HAD scale designed to screen for psychiatric morbidity. The aim was threefold: to determine the

*Received 17 November 1997*
*Revised 16 September 1998*
*Accepted 19 November 1998*

*Correspondence to:* E Hammerlid level of mental distress before, during and after treatment in head and neck cancer patients and the prevalence of probable mood disorder; to explore relationships between mental distress and tumour location, stage, performance status, age, sex and marital status; and to analyse the factor structure and stability of the HAD scale since the empirical evidence of a two-dimensional structure (anxiety and depression) is not unequivocal (Razavi et al, 1990; Brandberg et al, 1992).

### PATIENTS AND METHODS

### Study design

Patients were recruited from the University Hospitals of Göteborg, Malmö and Lund in Sweden, and the Norwegian Radium Hospital and the National Hospital in Oslo, Norway, in 1993–1994. All adult patients with an untreated primary head and neck cancer (ICD-9 141–148, 160, 161 and 196) were invited consecutively to participate in the study at all inclusion centres. Patients who were thought to be unable to answer the QL questionnaires due to senile dementia, mental disturbance or severe intercurrent disease were excluded, as were patients with lymphoma, malignant melanoma or skin cancer in the head and neck region. There was no limitation regarding age or performance status.

Patients answered the questionnaires at the time of diagnosis and then 1, 2, 3, 6 and 12 months after the treatment had started. The first questionnaire was given to the patients at the weekly tumour conference at each centre. The other questionnaires were mailed to the patients at the appropriate time. If the patients did not return the questionnaire within 10 days, they were reminded once. A completed questionnaire at diagnosis was a prerequisite for inclusion and was regarded as the patient's informed consent to inclusion. The study was approved by the local ethics committees.

At inclusion, the tumour location according to ICD-9, tumour, node and metastasis (TNM) classification (UICC, 1987), SNO med code for histopathology, planned treatment and curative or palliative intent were noted. The clinical data included co-morbidity, weight, height, weight loss (during the last 3 months), time of onset of tumour-related symptoms and Karnofsky Performance Status (KPS) (Karnofsky et al, 1948). After 13 months, current weight, KPS, treatment received and treatment response were noted together with present tumour status.

### QL questionnaires

#### *HAD scale*

The HAD scale, the focus of this paper, has been designed to screen for psychiatric morbidity in patients with somatic illness. It comprises two scales, one for depression (seven questions) and one for anxiety (seven questions). The questionnaire has been constructed so that somatic questions are avoided. Cut-offs have been established for when to regard a patient as a probable (> 10 points, on one scale) or possible (> 7, on one scale) case of psychiatric illness (Zigmond and Snaith, 1983).

The HAD scale has been extensively documented in patients with cancer in many countries and its validity has been examined in a variety of diagnostic groups (Aylard et al, 1987; Barczak et al, 1988; Razavi et al, 1990; Hopwood et al, 1991; Moorey et al, 1991; Caroll et al, 1993; Sullivan et al, 1993; Ibbotson et al, 1994). It has also been used in Scandinavia to screen for psychiatric illness among head and neck cancer patients (Hammerlid et al, 1997*a*, 1997*b*) as well as among patients with other types of cancer (Bergman et al, 1991; Brandberg et al, 1992, 1995; Nordin et al, 1996).

#### *Study-specific questionnaire*

This questionnaire contained eight self-reported questions relating to family, education, work and smoking habits.

### Patients

A total of 357 patients were included in the study; 111 patients in Oslo, 106 in Malmö/Lund and 140 in Göteborg. The recruitment period varied from 12 to 18 months due to local circumstances. The study population is described in Table 1. The mean age was 63 years (range 18–88) and the majority were males (72%). The most common subgroup of head and neck cancer ($n$ = 122) was oral cavity tumours (ICD 143 gingival; 144 floor of the mouth; 145 other oral and oral tongue). Eighty-nine patients had pharyngeal cancer (ICD 146 tonsils; 147 nasopharynx; 148 hypopharynx and tongue base carcinoma), 86 laryngeal carcinoma and 60 patients had 'other' tumour locations (ICD 142 salivary glands; 160 nose and sinuses; 196 unknown primary). At diagnosis, 46 patients (13%) were under treatment for hypertension or heart failure, 30 patients (8%) for pulmonary problems and 70 patients (20%) for another disease. Less than 10% had been treated for a previous malignancy ($n$ = 25).

**Table 1** Background treatment and follow-up data for the whole study group and selected subgroups

| | Total group | Male | Female | Oral cavity | Pharynx | Larynx | Other |
|---|---|---|---|---|---|---|---|
| Number | 357 | 256 (72%) | 101 (28%) | 122 | 89 | 86 | 60 |
| Females | 101 (28%) | | | 40 (33%) | 27 (30%) | 14 (16%) | 20 (33%) |
| Mean age | 63 | 63 | 61 | 62 | 59 | 66 | 65 |
| Stage[a] I+II | 141 (41%) | 103 (41%) | 38 (40%) | 56 (46%) | 14 (16%) | 55 (64%) | 16 (33%) |
| III+IV | 204 (59%) | 146 (59%) | 58 (60%) | 65 (54%) | 75 (84%) | 31 (36%) | 33 (67%) |
| Rt | 314 (88%) | 232 (89%) | 86 (85%) | 96 (79%) | 85 (96%) | 82 (95%) | 51 (85%) |
| BT | 56 (16%) | 39 (16%) | 17 (17%) | 29 (24%) | 24 (27%) | 0 | 7 (12%) |
| Chemo | 68 (19%) | 46 (18%) | 22 (22%) | 13 (11%) | 33 (37%) | 8 (6%) | 13 (22%) |
| Surgery | 133 (37%) | 86 (34%) | 47 (47%) | 79 (65%) | 10 (11%) | 7 (8%) | 37 (62%) |
| Relapse | 56 (16%) | 38 (15%) | 18 (18%) | 24 (20%) | 10 (11%) | 13 (15%) | 9 (15%) |
| Survival | 280 (78%) | 198 (76%) | 82 (81%) | 90 (74%) | 66 (74%) | 74 (86%) | 48 (80%) |

[a] Stage is missing for 12 patients: one gingival carcinoma, seven sinus and nose carcinoma, and four tumour colli. Rt: External radiation therapy; BT: Brachytherapy; Chemo: chemotherapy; Surgery: surgery towards the primary tumour; Relapse: relapse within the study-year; Survival: Survival rate after 1 year.

*© Cancer Research Campaign 1999*

### Sociodemographic data

At diagnosis, 62% of the patients were retired and 30% were working. The remainder were studying or unemployed. Thirty patients (9%) had children living in their household. Ninety-nine patients (28%) were living alone. More than half of the patients had only compulsory school education (58%) and the rest had college or university education.

### Treatment and follow-up

Treatment and follow-up after 1 year are shown in Table 1. The majority of patients had combined treatment; most of them had external radiation therapy. The mean radiation dose for the primary tumour was 60 Gy and the mean regional node dose was 46 Gy. Fifty-six patients received interstitial radiation therapy, all but two were treated in Göteborg.

A total of 133 patients (37%) underwent primary tumour surgery and 57 patients (16%) had a neck dissection. Sixty-eight patients (19%) were treated with chemotherapy, mainly in Göteborg. All but three patients were given two or three cycles of cisplatin (CDDP) in combination with 5-fluorouracil.

Of the 56 relapses, 33 were local, 22 regional and 14 patients had distant metastasis (13 patients had a combined local and regional relapse). No additional treatment was given in 15 cases; the others received salvage surgery ($n$ = 25), palliative chemotherapy ($n$ = 8) or radiation therapy ($n$ = 4) (information missing in four cases).

The mean KPS was 89 at the 1-year follow-up, compared to 92 at diagnosis (value 0–100). At the 1-year follow-up, 234 patients (65%) were alive and tumour-free without being treated for any relapse, 14 patients (4%) had been treated for one relapse but were tumour-free at the 1 year control, while 32 patients (9%) had an active tumour disease. Seventy-seven patients were dead, 50 (14%) due to the head and neck cancer, three patients (1%) due to another cancer, 14 patients (4%) due to another disease and ten patients (3%) had died of unknown causes. Thus, the overall survival rate after 1 year was 78%.

### Statistical analysis

For comparison between two groups, Fisher's non-parametric permutation test was applied and for analysing proportions between groups Fisher's exact test was used (Bradley, 1968*a*, 1968*b*). To test relationships between variables, Pitman's non-parametric permutation test (Bradley, 1968) was the preferred method. In order to adjust for confounding variables, a non-parametric partial correlation analysis based on Mantel's technique of pooling (Mantel, 1963) applied to Pitman's non-parametric permutation test was used. Pearson's correlation coefficient was used for descriptive purposes. The significance level was set at 5% throughout. A logistic regression analysis was performed to identify predictors of mental disturbance.

To test the two-dimensional structure of the HAD scale, principal components and common factor analysis (orthogonal rotation) were performed at diagnosis (Nunnally and Bernstein, 1994). Further, multitrait analysis was applied to test internal consistency (how much each question contributes to the anxiety or depression scale) and discriminant validity (how specific each anxiety question is in relation to the depression dimension and vice versa) (Nunnally and Bernstein, 1994). Cronbach's coefficient was reported as an estimate of the internal consistency reliability (Cronbach, 1951).

To avoid multiple significance testing, we selected three of the six measurement points: at diagnosis (before treatment), at 3 months (just after finishing treatment), when symptoms and problems are at their peak (Hammerlid et al, 1997*a*), and at the 1-year follow-up. The significance testing included only those patients who completed 3 months ($n$ = 261) and 12 months ($n$ = 215) follow-up respectively.

### RESULTS

Longitudinal data will be presented for the whole study group as well as for different subgroups of patients with respect to sex, age, tumour stage and tumour location.

### Compliance

Patients consecutively referred to the weekly tumour conferences at the different centres were asked to participate. A total of 357 patients agreed to participate and answered the first questionnaire (one questionnaire missing). Three hundred and six patients of 345 alive answered the second questionnaire one month after the start of treatment (89%), 290 of 330 (88%), surviving patients answered the third questionnaire 2 months after the start of treatment, 261 of 315 (83%) surviving patients answered the fourth questionnaire 3 months after the start of treatment, 239 of 309 (77%) the fifth questionnaire 6 months after the start of treatment and 215 of 280 (77%) patients alive answered the sixth questionnaire 1 year after the start of treatment.

### Questionnaire

#### *Performance of the questionnaire*

Few patients were omitted due to missing data ($n$ = 6, 1.7%, at diagnosis). The mean score was 4.75 for the anxiety scale and 3.8 for the depression scale. There were more floor than ceiling effects, i.e. 9% of the patients scored zero on the anxiety scale, compared to 15% on the depression scale. Only 0.3% of the patients achieved the maximum score on either of the scales. Cronbach's $\alpha$ (internal consistency) was 0.89 and 0.82 for the anxiety and depression scales respectively, i.e. well above the limit of 0.70. The Pearson correlation coefficient for each question versus the two scales showed that all questions in both scales correlated higher with their own scale, corrected for overlap, than with the other scale. The lowest within-scale correlation was found for 'I lost interest in my appearance' ($r$ = 0.37) (depression scale, corrected for overlap). The anxiety dimension showed better discriminant validity than the depression dimension, i.e. six of the seven anxiety questions versus three of seven reflecting depression correlated significantly higher with their own scale. The item 'I can sit at ease and feel relaxed' was unspecific, i.e. showed substantial correlation with both dimensions. The psychometric performance of the HAD scale seemed consistent over time since the same psychometric results were found at 3 and 12 months.

#### *Clinical results*

***Total study sample*** The *longitudinal* results from the HAD scale for the whole study population are shown in Table 2. The number of patients scoring as a probable or possible case of anxiety disorder was highest at diagnosis (32%), after which the

*© Cancer Research Campaign 1999*

**Table 2** Results from the HAD scale for all patients answering at each measurement point

| | Diagnosis | 1 month | 2 months | 3 months | 6 months | 12 months |
|---|---|---|---|---|---|---|
| Number of patients | 356 | 306 | 290 | 261 | 239 | 215 |
| Poss. Anxiety | 41 (12%) | 29 (9%) | 33 (11%) | 35 (13%) | 29 (12%) | 20 (9%) |
| Prob. Anxiety | 71 (20%) | 44 (14%) | 33 (11%) | 31 (12%) | 19 (8%) | 23 (11%) |
| Poss. Depr | 39 (11%) | 45 (15%) | 51 (18%) | 30 (11%) | 27 (11%) | 19 (9%) |
| Prob. Depr | 23 (6%) | 34 (11%) | 32 (11%) | 34 (13%) | 22 (9%) | 18 (8%) |
| No pts > 7 on a scale | 127 (36%) | 104 (34%) | 102 (35%) | 89 (34%) | 71 (30%) | 55 (26%) |

Questionnaire: Measurement point. Number of patients: number of patients answering the questionnaire. Poss. Anxiety: possible anxiety disorder, number of patients scoring 8–10 on the anxiety scale. Poss. Depr: possible depression disorder, number of patients scoring 8–10 on the depression scale. Prob. Anxiety: probable anxiety disorder, number of patients scoring 11 or more on the anxiety scale. Prob. Depr: probable depression disorder, number of patients scoring 11 or more on the depression scale. No pts > 7 on a scale: number of patients scoring > 7 on one scale; the patient is only counted once even if the scores exceed 7 on both scales. %: Percentage of the patients scoring > 7, on one scale.

**Table 3** Number of patients scoring better, worse or unchanged on the HAD scale between diagnosis and 3 months ($n$ = 260) and between diagnosis and 12 months ($n$ = 214)

| | 0–3 Months | | 0–12 Months | |
|---|---|---|---|---|
| | Anxiety | Depression | Anxiety | Depression |
| New poss. cases | 17 | 25 | 6 | 14 |
| New prob. cases | 13 | 26 | 4 | 14 |
| New poss. + prob. cases | 30 (12%) | 51 (20%) | 10 (5%) | 28 (13%) |
| 0–7 | 152 | 176 | 140 | 157 |
| 8–10 | 5 | 4 | 5 | 4 |
| 11+ | 18 | 8 | 19 | 4 |
| Unchanged score | 175 (67%) | 188 (72%) | 164 (77%) | 165 (77%) |
| Decrease of poss. cases | 23 | 15 | 18 | 14 |
| Decrease of prob. cases | 32 | 6 | 22 | 7 |
| Decrease of poss. + prob. cases | 55 (21%) | 21 (8%) | 40 (19%) | 21 (10%) |

Poss. case: possible case of anxiety or depression disorder, score 8–10; prob. case: probable case of anxiety or depression disorder, score 11+. New poss. cases: number of patients that deteriorated from scoring normally at diagnosis (0–7) to scoring as possible cases of anxiety or depression (8–10) at 3 months. New prob. cases: number of patients that deteriorated from scoring normally (0–7) or as a possible case of anxiety or depression (8–10) at diagnosis to scoring as a probable cases of anxiety or depression (11+) at 3 months. Unchanged score: number of patients that scored the same at both diagnosis and 3 months or diagnosis and 12 months. Decrease of poss. cases: number of patients that improved from scoring as a possible case of anxiety or depression to normal score. Decrease of prob. cases: number of patients that improved from scoring as a probable case of anxiety or depression to a possible case or normal score.

number slowly decreased. At the 1-year follow-up every fifth patient (20%) still scored above 7 on the anxiety scale. The highest number of patients scoring above 7 on the depression scale occurred 2 months after diagnosis (29%), i.e. during treatment, while the lowest number was seen at diagnosis and at the 1-year follow-up (17%). The number of patients scoring high on either of the scales was highest at diagnosis (36%) but did not change very much until the 1-year follow-up (26%). To find out if the changes during the year were significant, we selected three target measurement points out of six: at diagnosis, after finishing the treatment (3 months) and the follow-up at 12 months. The changes between diagnosis and 3 months ($n$ = 261) were significant for both possible and probable anxiety and depression ($P$ < 0.01), while between diagnosis and 12 months ($n$ = 215) only the change for probability of an anxiety disorder was significant ($P$ < 0.01) (data not shown).

At 3 and 12 months, we examined the proportion of patients that scored the same (0–7, 8–10, or 11+) or had improved/deteriorated compared to diagnosis (Table 3). The majority of the patients (58–75%) scored as non-cases (score 0–7) on the anxiety or depression scale at both 0–3 and 0–12 months. There were new possible and probable cases of psychiatric illness at both 3 and 12 months, the largest number being found on the depression scale at 3 months compared to diagnosis. The best improvement was found on the anxiety scale between diagnosis and 3 months.

### Subgroups of patients

Before any significance testing was performed between sex, stage, age group, KPS and HAD scale score, we examined potential relationships between background variables. We found no significant correlation between disease stage and age, between sex and age, or between stage and sex, but a strong correlation indicating that the lower the KPS, the more advanced was the tumour stage and the lower the KPS, the higher the patient's age (Pitman non-parametric correlation coefficients, $P$ < 0.001).

### Subgroups of patients at diagnosis

The HAD results for different subgroups at diagnosis are presented in Table 4. When comparing females with males, we

© Cancer Research Campaign 1999 

**Table 4** Results from the HAD scale at diagnosis for the whole study group and different subgroups for patients answering the questionnaire

| | Total group | Sex | | Age | | Stage | |
|---|---|---|---|---|---|---|---|
| | | Male | Female | –64 | 65+ | I+II | III+IV |
| Number of pts | 356 | 255 | 101 | 169 | 187 | 140 | 204 |
| Poss. Anxiety | 41 (12%) | 26 (10%) | 15 (15%) | 20 (12%) | 21 (11%) | 12 (9%) | 28 (14%) |
| Prob. Anxiety | 71 (20%) | 40 (16%) | 31 (31%) | 50 (30%) | 21 (11%) | 30 (21%) | 38 (19%) |
| Prob. + Poss. Anxiety | 112 (312%) | 66 (26%) | 46 (46%) | 70 (42%) | 42 (22%) | 42 (30%) | 66 (33%) |
| Poss. Depression | 39 (11%) | 24 (9%) | 15 (15%) | 21 (12%) | 17 (9%) | 15 (11%) | 22 (11%) |
| Prob. Depression | 23 (6%) | 16 (6%) | 7 (7%) | 9 (5%) | 14 (7%) | 5 (4%) | 18 (9%) |
| Prob. + Poss Depression | 62 (17%) | 40 (15%) | 22 (22%) | 30 (17%) | 31 (16%) | 20 (15%) | 40 (20%) |
| No pts > 7 on a scale | 127 (36%) | 78 (30%) | 49 (49%) | 72 (43%) | 55 (29%) | 46 (33%) | 77 (38%) |

| | Karnofsky | | | Tumour location | | | |
|---|---|---|---|---|---|---|---|
| | 100–90 | 80 | 70– | Oral | Pharynx | Larynx | Other |
| Number pts | 274 | 38 | 32 | 122 | 89 | 86 | 60 |
| Poss. Anxiety | 30 (11%) | 6 (16%) | 5 (16%) | 25 (20%) | 5 (6%) | 8 (9%) | 3 (5%) |
| Prob. Anxiety | 52 (19%) | 5 (13%) | 11 (34%) | 25 (20%) | 17 (19%) | 16 (19%) | 13 (22%) |
| Prob. + Poss. Anxiety | 82 (30%) | 11 (29%) | 16 (50%) | 50 (40%) | 22 (25%) | 24 (28%) | 16 (27%) |
| Poss. Depression | 28 (10%) | 5 (13%) | 4 (13%) | 12 (10%) | 11 (12%) | 13 (15%) | 3 (5%) |
| Prob. Depression | 14 (5%) | 3 (8%) | 6 (19%) | 8 (7%) | 8 (9%) | 4 (5%) | 3 (5%) |
| Prob. + Poss. Depression | 42 (15%) | 8 (21%) | 10 (32%) | 20 (17%) | 19 (21%) | 17 (20%) | 6 (10%) |
| No pts > 7 on a scale | 92 (34%) | 15 (39%) | 16 (50%) | 55 (45%) | 28 (31%) | 27 (31%) | 17 (28%) |

Poss. Anxiety: possible anxiety disorder, number of patients scoring 8–10 on the anxiety scale. Prob. Anxiety:probable anxiety disorder, number of patients scoring 11+ on the anxiety scale. Prob. + Poss. Anxiety: number of patients scoring as either a probable or possible case of anxiety. Poss. Depression: possible depression disorder, number of patients scoring 8–10 on the depression scale. Prob. Depression: probable depression disorder, number of patients scoring 11+ on the depression scale. Prob. + Poss. Depression: number of patients scoring as either a probable or possible case of depression. No pts > 7 on a scale: number of patients (%: percentage) scoring > 7 on one scale; the patient is only counted once even if the scores exceed 7 on both scales.

**Table 5** The HAD scale and subgroupings by sex, age, stage and performance status at three measurement points

| | Diagnosis (*n* = 359) | 3 months (*n* = 260) | 12 months (*n* = 214) |
|---|---|---|---|
| Sex | Anxiety[b]<br>Depression<br>> 7 on a scale[b] | Anxiety<br>Depression<br>> 7 on a scale | Anxiety[a]<br>Depression<br>> 7 on a scale |
| Age<br>–64 vs. >65 | Anxiety[b]<br>Depression<br>> 7 on a scale[a] | Anxiety<br>Depression<br>> 7 on a scale | Anxiety[b]<br>Depression<br>> 7 on a scale |
| Stage<br>I+II vs. III+IV | Anxiety<br>Depression<br>> 7 on a scale | Anxiety[a]<br>Depression<br>> 7 on a scale | Anxiety<br>Depression<br>> 7 on a scale |
| Karnofsky | Anxiety<br>Depression[b]<br>> 7 on a scale | Anxiety<br>Depression[a]<br>> 7 on a scale[a] | Anxiety<br>Depression[a]<br>> 7 on a scale |

Diagnosis, 3 months and 12 months: measurement points. The Table shows the significant differences in probability of anxiety and depression between the sexes, age groups and stages, and the significant correlation between Karnofsky's performance status and the HAD. >7 on a scale means the number of patient scoring above 7 points on either anxiety or depression. [a]$P < 0.05$), [b]$P < 0.01$.

found a significantly higher prevalence of anxiety disorder for females ($P < 0.01$). Further, they scored above 7 points on either of the scales significantly more often ($P < 0.01$).

When patients before retirement were compared with patients after retirement, the younger patients reported significantly more anxiety ($P < 0.01$) and significantly more often scored above seven on either of the scales ($P < 0.05$). This result was still valid after adjusting for KPS.

Patients with advanced disease (stage III+IV) more often scored as a possible or probable case of depression than patients with small tumours (stage I+II) but the difference was not significant (20% vs 14%). There was a significant correlation between KPS and depression ($P < 0.01$) but not anxiety.

We did not find any significant difference when the tumour locations were compared; the oral cavity tumour patients scored above seven on either of the scales in 45% of the cases, compared to 31% of the pharyngeal and laryngeal cancer patients and 28% of those with ‘other tumours’.

We also tested if people living alone ($n = 99$) were at greater risk of developing mental distress levels approaching psychiatric morbidity than patients living with someone ($n = 253$), since inadequate social support might be considered detrimental. People living alone scored higher for ‘probable cases of depression’ (9% versus 5%) and for ‘possible cases of anxiety’ (13% versus 6%) but the differences were not significant. There was no significant difference, at diagnosis, in the occurrence of anxiety or depression

 *© Cancer Research Campaign 1999*

disorder between the group of patients interrupting the study and the group completing the study.

### Longitudinal results for subgroups of patients

Since there were differences at diagnosis in the HAD score depending on sex, age group, stage and KPS, we decided to examine if there were any significant differences between these subgroups at other time points. The HAD scores at three measurement points were selected: at diagnosis, at 3 months and at 12 months. The numbers of patients scoring as probable or possible cases of anxiety or depression are displayed in Figures 1–4 and the significant differences between subgroups are shown in Table 5.

Female patients more often scored as probable or possible cases of anxiety during the study-year (Figure 1A), the difference at diagnosis and 12 months being significant (Table 5). There was no significant difference in prevalence of depression between the sexes (Figure 1B and Table 5).

Patients before retirement were more anxious than patients after retirement at all measurement points (Figure 2A) and the differences were significant at diagnosis and at 12 months (Table 5), while there was no consistent difference between the two age groups when comparing the number of patients scoring as probable or possible cases of depression. Patients with large tumours more often scored as probable or possible cases of anxiety and depression compared with patients with small tumours (Figure 3A,B) but there was only one significant difference (anxiety 3 months). Patients with low KPS were more often depressed (Figure 4B) and the difference at diagnosis and at 12 months was significant (Table 5).

In order to identify risk factors for developing mental distress or psychiatric morbidity, a logistic regression analysis was performed where age, sex, tumour site, tumour stage, KPS, living alone or not and HAD score at baseline (possible or probable cases of anxiety or depression) were considered potential predictors. The only predictor found for psychiatric disturbence at twelve months was probable and/or possible anxiety or depression at diagnosis.

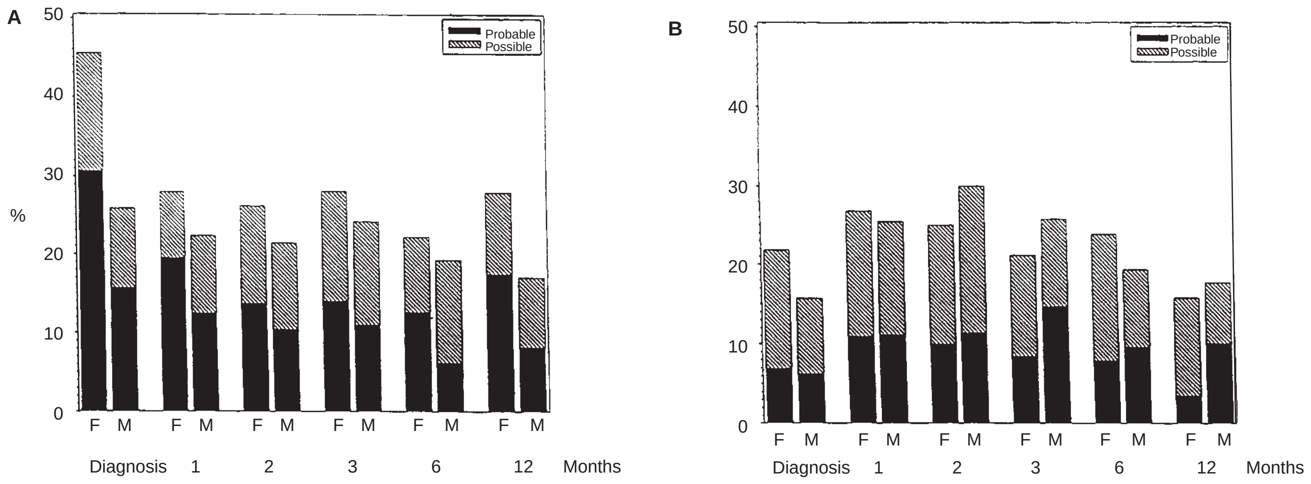

**Figure 1** The percentage of females (F) versus males (M) who scored as a possible or probable case of anxiety (**A**) or depression (**B**) at the different measurement points during the study year.

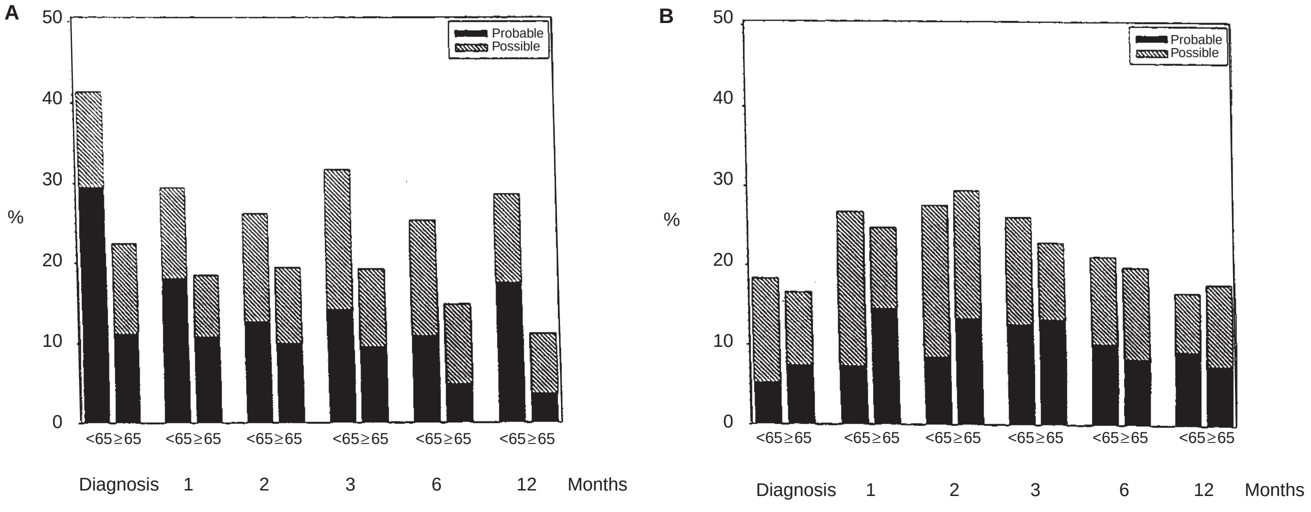

**Figure 2** The percentage of patients, before and after retirement, who scored as a possible or probable case of anxiety (**A**) or depression (**B**) at the different measurement points during the study year. < 65 years (before retirement) versus patients 65 years or older (after retirement)

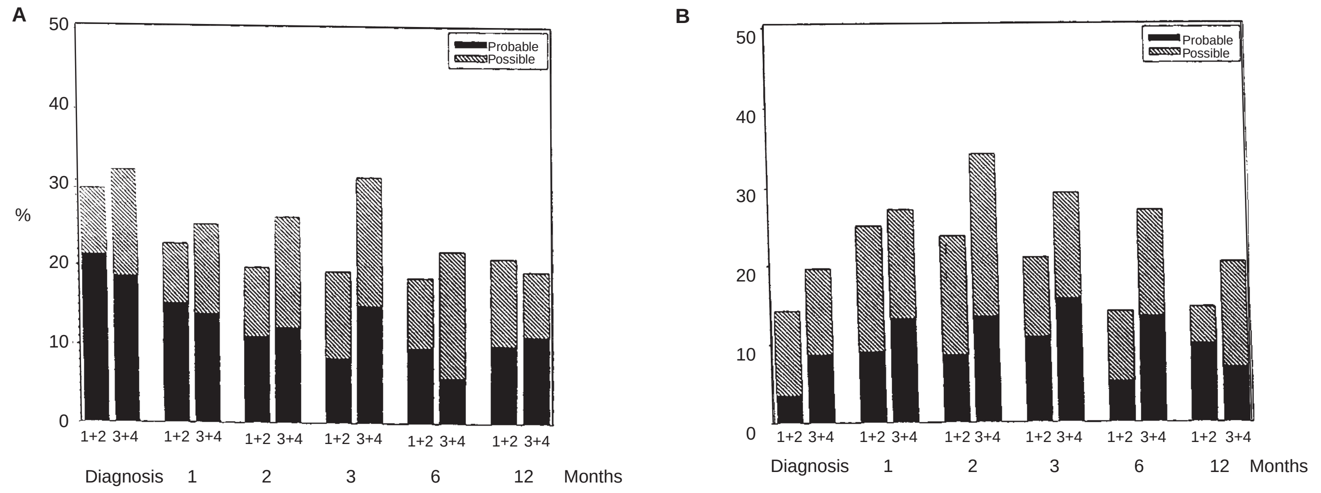

**Figure 3** The percentage of patients who scored as a possible or probable case of anxiety (**A**) or depression (**B**) at the different measurement points during the study year by stage. 1+2 = stage I+II and 3+4 = stage III+IV

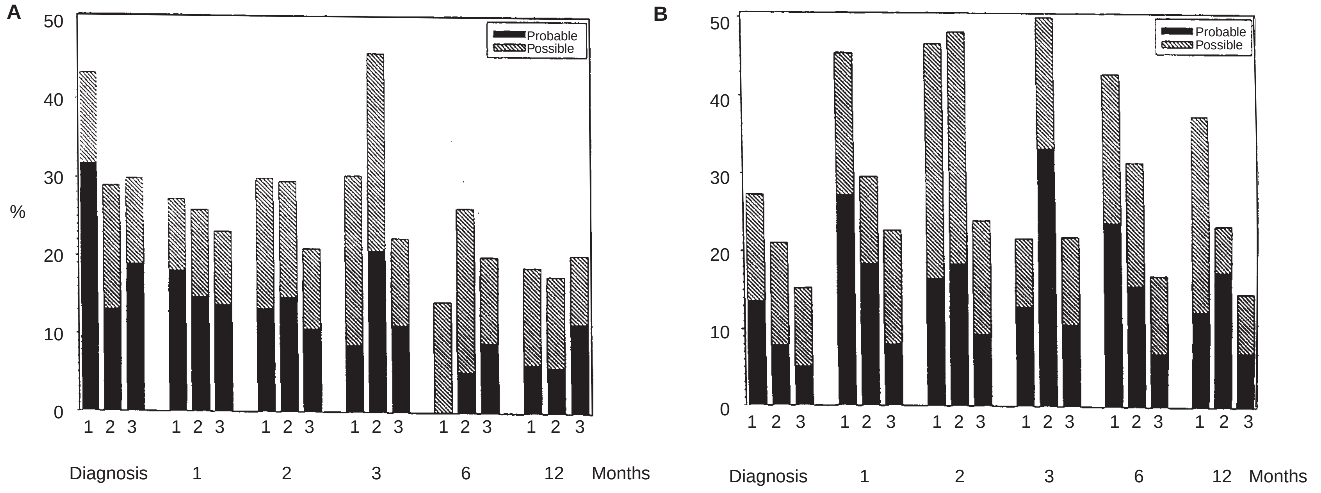

**Figure 4** The percentage of patients who scored as a possible or probable case of anxiety (**A**) or depression (**B**) at the different measurement points during the study year by Karnofsky's performance status (KPS). 1 = KPS –70, 2 = KPS 80, 3 = KPS 90–100

### DISCUSSION

This head and neck study sample was considered representative of such patients in Sweden and Norway since a high percentage of the patients that fulfilled the inclusion criteria were included (Hammerlid et al, unpublished data) and the compliance rate was high (77%). Compliance was worse for patients with an active disease (56%) than for patients without tumour (80%) at the 1-year follow-up. The results therefore probably underestimate the true prevalence of psychiatric morbidity 1 year after diagnosis of head and neck cancer since the results from this study have shown that emotional distress and psychiatric morbidity are more common among patients with low KPS and more advanced disease. We excluded patients not able to answer the QL questionnaires and patients with mental disturbance. However, not one patient was excluded due to mental disturbance when we examined the reasons for exclusion in one of the centres. We therefore consider this exclusion criterion to be a minor problem.

The anxiety and depression scales displayed different 'patterns' over time. We found the highest number of patients with probable or possible anxiety disorder at diagnosis, while the highest number of patients with depression was found during treatment. There were new cases of possible or probable mood disorder at each measurement point (Table 3). We therefore conclude that it is useful to include the HAD scale not only at diagnosis but repeatedly during the first year of treatment. It will thereby be possible to identify patients in need of psychiatric consultation and treatment continuously.

Females scored markedly worse than males on the anxiety scale at diagnosis and at the one-year follow-up but not significantly so at the other measurement points (Figure 1A). The same results were found in two studies of psychological distress in mixed cancer populations. In one study sample the patients received radiation therapy (Maher et al, 1996) and in the other patients were compared at different stages of their disease (Caroll et al, 1993). A higher level of emotional distress for females was also found in a study among gastrointestinal cancer patients (Nordin et al, 1996). The results presented here are in line with the significantly lower emotional functioning scores for females at diagnosis found in the same study sample, using the EORTC QLQ-C30 (Hammerlid et al,

 *© Cancer Research Campaign 1999*

unpublished data). It therefore seems likely that females are more worried than males initially in the course of their cancer disease, but not to the same extent over time. This finding is in contrast, at least partly, to a general tendency in the literature that women report more symptoms/problems than men (Ware et al, 1993).

Advanced stage and low performance status seemed to be the major determinants of a high score on the HAD scales. Partial correlation analyses of stage, KPS and HAD pointed to the Karnofsky's performance status as the stronger predictor. Patients with an advanced disease more often receive combined treatment than patients with small tumours. The treatment period is therefore longer and the side-effects greater, which may influence the level of psychiatric distress. We found that patients with a more advanced disease (stage III+IV) reported more depression (Figure 3B), but the strongest correlation between psychiatric distress and the other tested variables was found for KPS (Figure 4), which corresponds to findings by others, i.e. the lower the performance status, the higher the level of psychological distress (Razavi et al, 1990; Kaasa et al, 1993). Unfortunately, physicians do not pay enough attention to the probability of psychiatric disorders among advanced head and neck cancer patients. This may be due to lack of training and insufficient knowledge of the psychological aspects of the disease (Maguire, 1985).

Patients before retirement scored worse than patients after retirement at diagnosis, and a correlation between age and the level of mental distress was found at diagnosis and at the 1 year follow-up (Figure 2). This result corresponded with an earlier finding of poorer emotional functioning in younger patients according to the EORTC QLQ-C30 (Hammerlid et al, unpublished data) at diagnosis. Further, a recent study of patients with advanced malignancies supports our results (Coates et al, 1997). The psychometric analyses, at three time points, supported the two-dimensional structure and stability of the HAD scale, in agreement with previous results (Moorey, Greer et al. 1991).

The potential risk factors identified for development of psychiatric morbidity among head and neck cancer patients in this study should increase health care providers' awareness of which patients are more likely to develop high levels of mental distress. This is of value since early detection and treatment of psychiatric illness has been found to improve outcome (Maguire et al, 1980), as has targeted intervention for patients at high risk (Moorey et al, 1994).

### CONCLUSIONS

We conclude that the HAD scale is a useful instrument for easy and cheap screening for psychiatric morbidity in head and neck cancer patients. In order to find all patients at risk of developing psychiatric disorder, it has to be used repeatedly since there were new cases of psychiatric morbidity at all measurement points. The number of patients with increased levels of mental distress was high throughout the study, anxiety being most common at diagnosis, while depression was most common during treatment. A low performance status and an advanced disease are stronger risk factors for mental distress than sex and age. The prevalence of psychiatric morbidity found in this study emphasizes the importance of improved diagnosis and treatment.

### ACKNOWLEDGEMENTS

This study was made possible by grants from the Nordic Cancer Union (project number NF-43258), the Swedish Cancer Society, the Assar Gabrielsson Foundation, King Gustav V, Jubilee Clinic Cancer Research Foundation in Göteborg, Cancer och Trafikskadades Riksförbund, the Göteborg Medical Society and the Medical Faculty, Göteborg University. Special thanks to Marita Hellqvist for secretarial assistance, to Lena Hörnestam for assistance with the collection of data at Sahlgrenska University Hospital, to Nils-Gunnar Pehrsson for statistical advice, to the Clinical Trial Office at the Radium Hospital (Chairman Einar Hannisdal), which was responsible for mailing the questionnaires to the Norwegian patients, and to Bente Moldaunet, who entered the data in Norway, to Monica Saxnes, who recruited the patients at the University Hospital in Lund, and to Bodil Rönnow, who entered the data in Malmö and Lund.

### REFERENCES

Aylard PR, Gooding JH (1987) A validation study of three anxiety and depression self-assessment scales. *J Psychosom Res* **31**: 261–268

Baile WF, Gibertini M (1992) Depression and tumor stage in cancer of head and neck. *Psycho-Oncology* **1**: 15–24

Barczak P, Kane N (1988) Patterns of psychiatric morbidity in a genito-urinary clinic. A validation of the Hospital Anxiety Depression Scale (HAD). *Br J Psychiatry* **152**: 698–700

Bergman B, Sullivan M (1991) Quality of life during chemotherapy for small cell lung cancer. *Acta Oncol* **30**: 947–957

Bjordal K and Kaasa S (1995) Psychological distress in head and neck cancer patients 7–11 years after curative treatment. *Br J Cancer* **71**: 592–597

Bjordal K, Kaasa S (1993) Quality of life of patients with head and neck cancer. Protocol for prospective Scandinavian multi-centre study. *Clin Otolaryngol* **18**: 72–79

Bradley JV (1968*a*). *Distribution-Free Statistical Tests*, pp. 78–80. Prentice-Hall: London

Bradley JV (1968*b*). *Distribution-Free Statistical Tests*, pp. 73–76. Prentice-Hall: London

Brandberg Y, Bolund C (1992) Anxiety and depressive symptoms at different stages of malignant melanoma. *Psycho-Oncology* **1**: 71–78

Brandberg Y, Månsson-Brahme E (1995) Psychological reactions in patients with malignant melanoma. *Eur J Cancer* **31A**: 157–162

Caroll BT, Kathol RG (1993) Screening for depression and anxiety in cancer patients using the Hospital Anxiety and Depression scale. *General Hospital Psychiatry* **15**: 69–74

Coates A, Porzolt F (1997) Quality of life in oncology practise: prognostic values of EORTC QLQ-C30 scores in patients with advanced malignancy. *Eur J Cancer* **33**: 1025–1030

Cronbach LJ (1951) Coefficient alpha and the internal structure of test. *Psychometrica* **16**: 297

Davies ADM, Davies C (1986) Depression and anxiety in patients undergoing diagnosticinvestigation for head and neck cancers. *Br J Psychiatry* **149**: 491–493

Espie CA, Freedlander E (1989) Psychological distress at follow-up after major surgery for intra-oral cancer. *J Psychosom Res* **33**: 441–448

Greer S (1994) Psycho-oncology: its aims, achievement and future tasks. *Psycho-oncology* **3**: 87–101

Hammerlid E, Bjordal K (1997*a*) A prospective longitudinal quality of life study of patients with head and neck cancer. *Otolaryngol Head Neck Surg* **116**: 666–673

Hammerlid E, Mercke C (1997*b*) A prospective quality of life study of patients with oral or pharyngeal carcinoma treated with external beam irradiation with or without brachytherapy. *Eur J Cancer Oral Oncol* **33**: 189–196

Harrison J and Maguire P (1994) Predictors of psychiatric morbidity in cancer patients. *Br J Psychiatry* **165**: 593–598

Harrison J, Maguire P (1994) Concerns, confiding and psychiatric disorder in newly diagnosed cancer patients: a descriptive study. *Psycho-oncology* **3**: 173–179

Hopwood P, Howell A (1991) Screening for psychiatric morbidity in patients with advanced breast cancer: validation of two self-report questionnaires. *Br J Cancer* **64**: 353–356

Ibbotson T, Maguire P (1994) Screening for anxiety and depression in cancer patients: the effects of disease and treatment. *Eur J Cancer* **30A**: 37–40

Kaasa S, Malt U (1993) Psychosocial distress in cancer patients with advanced disease. *Radiother Oncol* **27**: 193–197

Karnofsky DA, Abelman WH (1948) The use of nitrogen mustards in the palliative treatment of carcinoma. *Cancer* **1**: 634–656

© Cancer Research Campaign 1999

McDonough EM, Boyd JH (1996) Relationship between psychological status and compliance in a sample of patients treated for cancer of the head and neck. *Head Neck* **18**: 269–276

McQuellon RP and Hurt GJ (1997) The psychosocial impact of the diagnosis and treatment of laryngeal cancer. *Otolaryng Clin N Am* **2**: 231–241

Maguire P (1985) Improving the detection of psychiatric problems in cancer patients. *Soc Sci Med* **20**: 819–823

Maguire P (1992) Improving the recognition and treatment of psychiatric morbidity on a medical oncology ward. In *Recent Advances in Clinical Psychology,* Granville-Grossman K (ed). Churchill Livingstone: Edinburgh

Maguire P, Tait A (1980) Psychatric morbidity and physical toxicity associated with adjuvant chemotherapy after mastectomy. *Br Med J* **281**: 1179–1180

Maher EJ, Mackenzie C (1996) The use of the Hospital Anxiety and Depression Scale (HADS) and the EORTC QLQ-C30 questionnaires to screen for treatable unmet needs in patients attending routinely for radiotherapy. *Cancer Treat Rev* **22**: 123–129

Mantel N (1963) Chi-square test with one degree of freedom. Extension of the Mantel Haenszel procedure. *J Am Stat Assoc* **59**: 690–700

Moorey S, Greer S (1991) The factor structure and factor stability of the Hospital Anxiety and Depression Scale in patients with cancer. *Br J Psychiatry* **158**: 255–259

Moorey S, Greer S (1994) Adjutant psychological therapy for patients with cancer: outcome at one year. *Psycho-oncology* **3**: 39–46

Morton RP, Davies ADM (1984) Quality of life in treated head and neck cancer patients: a preliminary report. *Clin Otolaryngol* **9**: 181–185

Nordin K, Glimelius B (1996) Anxiety, depression and worry in gastrointestinal cancer patients attending medical follow-up control visits. *Acta Oncologica* **35**: 411–416

Nunnally JC and Bernstein IH (1994) *Psychometric Theory*. McGraw-Hill: New York

Rapoport Y, Kreitler S (1993) Psychosocial problems in head-and-neck cancer patients and their change with time since diagnosis. *Ann Oncol* **4**: 69–73

Razavi D, Delvaux N (1990) Screening for adjustment disorders and major depressive disorders in cancer in-patients. *Br J Psychiatry* **158**: 255–259

Shapiro PA and Kornfeld DS (1987) Psychiatric aspects of head and neck cancer surgery. *Psychiatry Clin North Am* **10**: 87–101

Sullivan M, Karlsson J (1993) Swedish obese subjects (SOS) – an intervention study of obesity. Baseline evaluation of health and psychosocial functioning in the first 1743 subjects examined. *Int J Obes* **17**: 503–512

Ware JE, Snow KK (1993) SF-36 *Health Survey Manual and Interpretation Guide*. New England Medical Center, The Health Institute: Boston

Westin T, Jansson A (1988) Mental depression is associated with malnutrition in patients with head and neck cancer. *Arch Otolaryngol Head Neck Surg* **114**: 1449–1453

Zigmond AS and Snaith RP (1983) The Hospital Anxiety and Depression Scale. *Acta Psychiatr Scand* **67**: 361–370

*© Cancer Research Campaign 1999*
